# Indirect excitation of ultrafast demagnetization

**DOI:** 10.1038/srep18970

**Published:** 2016-01-06

**Authors:** Boris Vodungbo, Bahrati Tudu, Jonathan Perron, Renaud Delaunay, Leonard Müller, Magnus H. Berntsen, Gerhard Grübel, Grégory Malinowski, Christian Weier, Julien Gautier, Guillaume Lambert, Philippe Zeitoun, Christian Gutt, Emmanuelle Jal, Alexander H. Reid, Patrick W. Granitzka, Nicolas Jaouen, Georgi L. Dakovski, Stefan Moeller, Michael P. Minitti, Ankush Mitra, Sebastian Carron, Bastian Pfau, Clemens von Korff Schmising, Michael Schneider, Stefan Eisebitt, Jan Lüning

**Affiliations:** 1 Sorbone Universités, UPMC Univ Paris 06, UMR 7614, LCPMR, 75005 Paris, France; 2 CNRS, UMR 7614, LCPMR, 75005 Paris, France; 3 Deutsches Elektronen-Synchrotron (DESY), Notkestraße 85, 22607 Hamburg, Germany; 4 The Hamburg Centre for Ultrafast Imaging, Luruper Chaussee 149, 22761 Hamburg, Germany; 5 Institut Jean Lamour, UMR CNRS 7198 – Université de Lorraine – boulevard des aiguillettes BP 70239, Vandoeuvre cedex F-54506 France; 6 Forschungszentrum Jülich GmbH, Peter Grünberg Institut (PGI-6), JARA- FIT, 52425 Jülich, Germany; 7 LOA, ENSTA ParisTech, CNRS, Ecole polytechnique, Université Paris-Saclay, 828 bd des Maréchaux, 91762 Palaiseau cedex France; 8 Department Physik, Universität Siegen, Walter-Flex-Str. 3, 57072 Siegen, Germany; 9 Stanford Institute for Materials and Energy Sciences, SLAC National Accelerator Laboratory, 2575 Sand Hill Road, Menlo Park, CA 94025, USA; 10 Van der Waals-Zeeman Institute, University of Amsterdam, 1018XE Amsterdam, The Netherlands; 11 Synchrotron SOLEIL, Saint-Aubin, Boîte Postale 48, 91192 Gif-sur-Yvette Cedex, France; 12 Linac Coherent Light Source, SLAC National Accelerator Laboratory, 2575 Sand Hill Road, Menlo Park, CA 94025, USA; 13 Division of Synchrotron Radiation Research, Lund University, Box 118, 22100 Lund, Sweden; 14 Institut für Optik und Atomare Physik, Technische Universität Berlin, 10623 Berlin, Germany

## Abstract

Does the excitation of ultrafast magnetization require direct interaction between the photons of the optical pump pulse and the magnetic layer? Here, we demonstrate unambiguously that this is not the case. For this we have studied the magnetization dynamics of a ferromagnetic cobalt/palladium multilayer capped by an IR-opaque aluminum layer. Upon excitation with an intense femtosecond-short IR laser pulse, the film exhibits the classical ultrafast demagnetization phenomenon although only a negligible number of IR photons penetrate the aluminum layer. In comparison with an uncapped cobalt/palladium reference film, the initial demagnetization of the capped film occurs with a delayed onset and at a slower rate. Both observations are qualitatively in line with energy transport from the aluminum layer into the underlying magnetic film by the excited, hot electrons of the aluminum film. Our data thus confirm recent theoretical predictions.

Ultrafast demagnetization is a very intriguing phenomenon. The existence and origin of this rapid loss of a thin film’s magnetization on the femtosecond timescale has been controversially debated ever since its discovery in 1996^1^. Recently, it has been proposed that the origin of this magnetization loss could be due to spin-dependent motion of the optically excited hot valence electrons causing a spatial redistribution of the magnetization, either to an adjacent metallic layer^2^,^3^ or within the magnetic layer itself^4^. Strong experimental evidence for this so-called superdiffusive spin transport has been found lately^5^,^6^,^7^,^8^.

However, the experiment of Eschenlohr and co-workers^8^, similar to our study reported here, has been challenged recently. Khorsand and co-workers^9^ showed that the Au cap layer employed in that experiment does not yield the postulated, and for the interpretation of the data crucial, attenuation of the incident IR pump pulse. Implementing a truly IR-opaque capping layer, we evade the limitation affecting the interpretation of the results of Eschenlohr and co-workers^8^. Proper characterization of the number of photons reaching the magnetic layer demonstrates that the transmitted intensity is by orders of magnitude too small to excite the ultrafast demagnetization process.

The experiment has been realized at the SXR instrument of the X-ray Free Electron laser LCLS^10^ equipped with the pnCCD camera^11^ using resonant magnetic small-angle scattering as probe technique for the temporal evolution of the local magnetization within the domains of a thin Co/Pd multilayer film. The data obtained undoubtedly prove that the ultrafast demagnetization process can be triggered without any direct interaction between the photons of the optical pump pulse and the magnetic layer. In addition, the data clearly resolve the presence of a delayed onset of this process as well as its slower evolution with respect to the dynamics directly induced by the IR pump pulse. We note that both these observations are consistent with an excitation process by hot electrons as proposed by Eschenlohr and co-workers^8^. However, and contrary to these authors, we think that those observations do not unambiguously prove that part of the magnetization is transferred to the metallic cap layer via superdiffusive spin transport^3^.

## Results

### Samples fabrication and characterization

**Figure 1:**
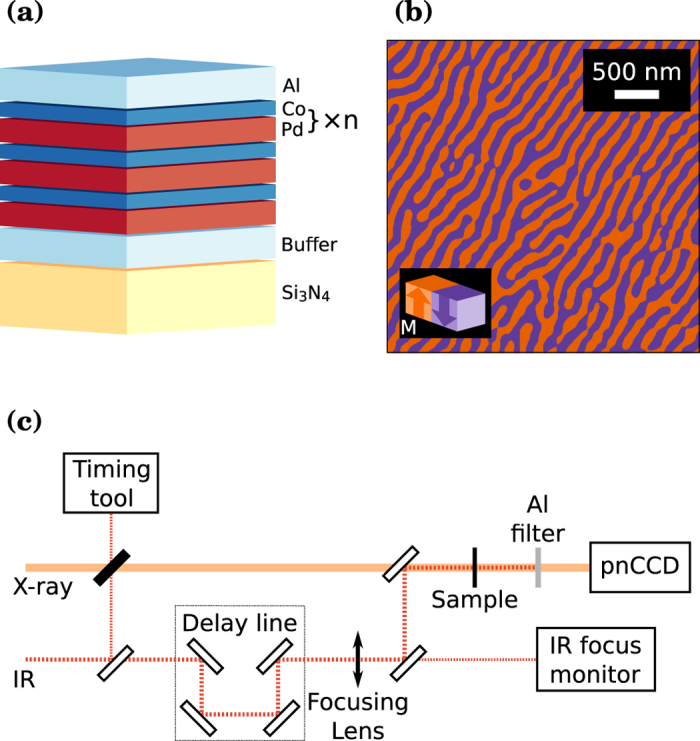
(**a**) Sketch of the multilayer composition exhibiting magnetic out-of-plane anisotropy. A magnetic domain structure exhibiting aligned stripe domains as shown by the MFM image in (**b**) is prepared using an oscillatory demagnetization procedure^22^. (**c**) Illustration of the experimental setup highlighting the optical IR – X-ray pulse cross-correlator for arrival time characterization, the positioning of the IR delay line unit, the out-of-vacuum IR focusing lens and the co-linear IR in-coupling. Not shown is the SXR monochromator, which was tuned to the Co L_3_ resonance at 778 eV.

The thin-film samples studied in this experiment have been grown by DC magnetron sputtering on 50 nm thin X-ray transparent Si_3_N_4_ membranes (see Fig. 1(a)). Two ferromagnetic 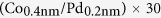 multilayer films were grown. One film was capped with a 3 nm thin Al layer to prevent oxidation of the magnetic multilayer. Since about 1.5 nm of this Al cap is instantaneously oxidized^12^, we refer to this film as *uncapped* Co/Pd film in the following. This is in contrast to the *capped* Co/Pd film on top of which a 40 nm thick Al film was grown.

To verify that this Al cap layer is indeed opaque for the IR pump pulse, a 40 nm thick Al layer has been grown on the same type of Si_3_N_4_ substrate. The transmission of this sample was measured to be 1.3 × 10^−4^ (sensitivity of the measurement has been 10^−6^). We will see below that this value corresponds to an Al opacity nearly two orders of magnitude larger than what is needed to exclude any direct IR photon excitation as origin of the observed demagnetization dynamics. Having also measured the IR reflectivity and transmission of the films, we can calculate the relative amount of pump pulse energy absorbed in the cap and magnetic layers of these films (Table 1). Finally, we can deduce from these data an absorption length of about 5.5 nm for the 800 nm IR light in Al, which is in line with values given in the literature^13^.

### Infrared pump soft X-ray probe experiment

**Figure 2:**
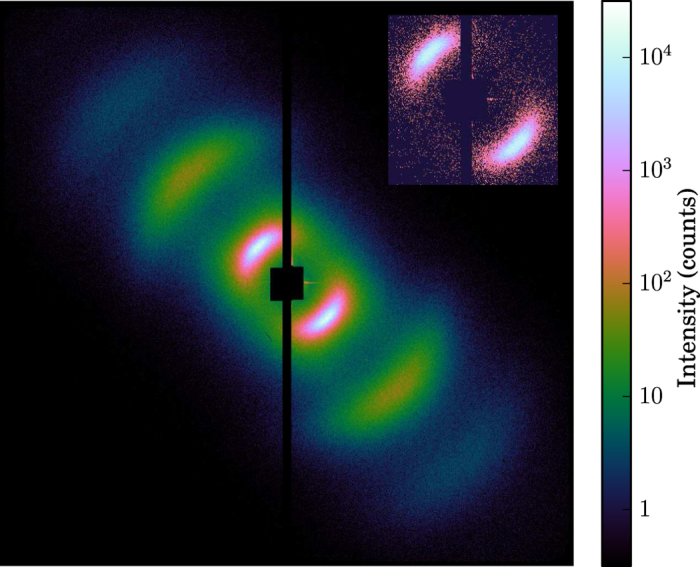
Resonant magnetic scattering pattern recorded at the Co L_3_ edge (778 eV). The pattern in inset has been recorded with a single X-ray pulse. The mean of about thousand such single shots pattern reveals scattering up to the fifth grating order (even orders are suppressed since up and down magnetic domains have the same size distribution), which reflects the high degree of alignment of the magnetic stripe domains.

As in previous experiments^5^,^14^, we have employed resonant magnetic small-angle X-ray scattering as probe technique, which characterizes in the presence of a magnetic domain structure a material’s local magnetization with nanometer spatial resolution. Prior to the experiment, a demagnetization procedure with an oscillating, successively decreasing magnetic field oriented parallel to the film surface has been employed to obtain a magnetic domain structure of well-aligned stripe domains^15^ (see Fig. 1(b)). Tuning the X-ray photon energy to the magnetically dichroic Co L_3_ edge, this grating-like magnetic domain structure gives rise to localized scattering intensities corresponding to the grating’s positive and negative diffraction orders. The clear visibility of up to the 5^*th*^ scattering order in the pattern reproduced in Fig. 2 indicates the high degree of domain alignment present in these films.

**Figure 3:**
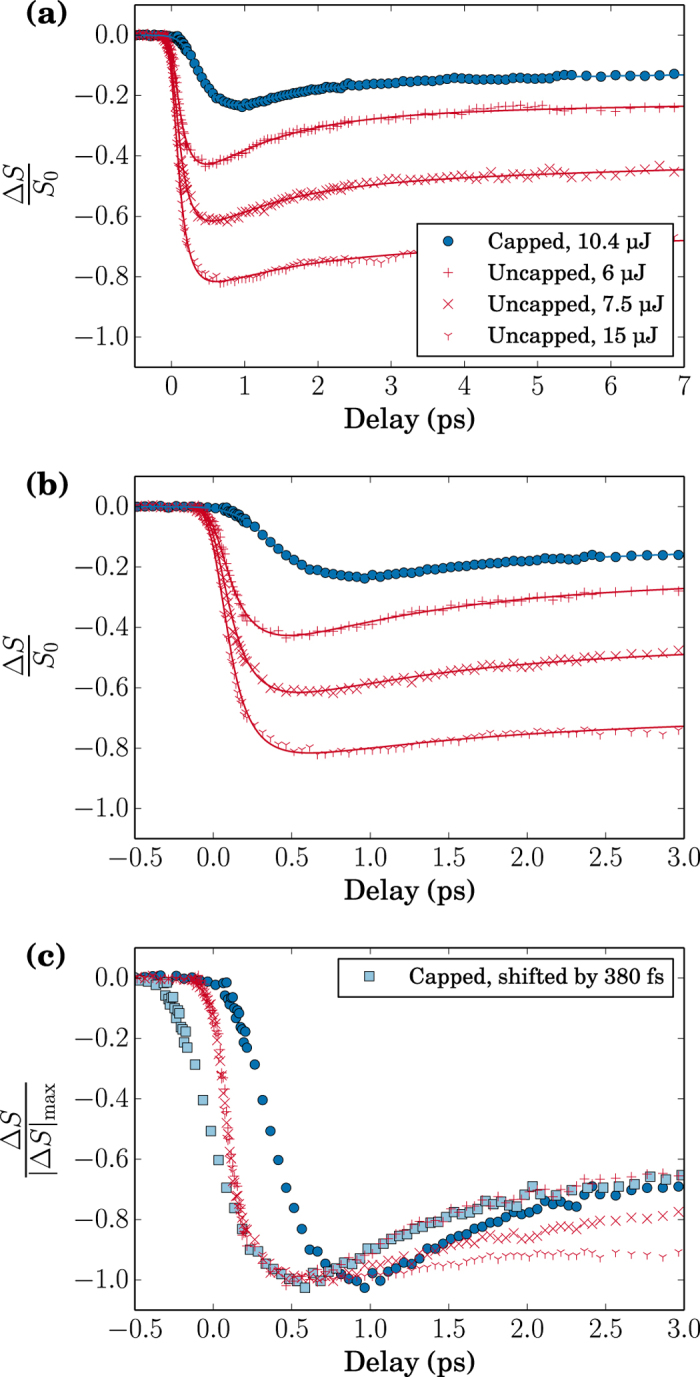
Magnetic scattering as a function of delay for capped (blue symbols) and uncapped (red symbols) samples at different pump fluences on a (**a**) long and (**b**) short timescale and (**c**) normalized to the maximum demagnetization. The solid lines are the best fit obtained for each curve. The demagnetization of the capped magnetic film is delayed compared to the uncapped film and also appears to be slower. The shifted curve of the capped film (light blue squares) clearly demonstrates these two points. It also shows that the partial recovery of this curve is similar to the one of the uncapped sample when weakly pumped.

The resonantly excited scattering pattern originating from a single X-ray pulse shown in the inset of Fig. 2 is representative for the ones recorded on both, the uncapped and the capped Co/Pd multilayer film. For this pattern recorded with negative pump probe delay (X-rays first), we see that the number of CCD counts within each of the two first order scattering peaks (roughly from 2 × 10^2^ to 10^4^ counts) is well above the noise level of the detector (a few counts). Such scattering patterns have been recorded for different IR pump fluence values while changing the IR-pump - X-ray probe delay continuously as discussed above. The magnetic scattering intensity is extracted from these pattern by integration of the plus and minus first order scattering peaks. Note that the scattering intensity is proportional to the square of the local magnetization within the domains^5^,^14^. Plotting these scattering intensities against the IR-pump – X-ray probe delay yields the curves shown in Fig. 3(a), which characterize the temporal evolution of the magnetization of the two Co/Pd films as a function of IR pump fluence. On a first glance one notes that the magnetization exhibits in all cases the typical behavior associated with the ultrafast demagnetization phenomenon^1^: a drastic drop of the magnetization occurring on a sub-picosecond time scale, which is followed by a partial magnetization recovery taking place with a slower time constant on the order of one to a few picoseconds. On the other hand, the comparison of the two films reveals that the onset of the magnetization dynamics of the capped film is clearly delayed with respect to the one of the uncapped film. We note that this delayed onset is the expected signature of the demagnetization dynamics induced by the hot electrons generated by the IR pump pulse in the metallic capping layer. This point is further discussed below.

The demagnetization curves of the uncapped Co/Pd film were excited with IR pump pulse energies of 20 μJ, 25 μJ and 50 μJ. Taking the reflectivity and absorption values from Table 1 we can derive values for the IR absorbed energy: 6 μJ, 7.5 μJ and 15 μJ respectively, giving rise to a maximum degree of demagnetization at about 0.5 ps delay of 24%, 38% and 57%. It has to be noted that this absorbed energy values might not correspond to the actual absorbed energy since the exact IR transmission through the transport line was not measured accurately. However, these values give us a very good relative scale to compare with the absorbed energy in the capped sample. Furthermore, the pump beam profile at the sample position was not measured with a sufficiently high accuracy to obtain an absolute fluence value. However, previous measurements on similar samples^14^,^16^ show that the fluences reached here should approximately be in the range of 5 to 15 mJ cm^−2^.

In case of the capped Co/Pd film, the IR pump pulse energy needed to be increased to 65 μJ to reach a demagnetization of 12%. Taking the film’s reflectivity and the layers’ absorption from Table 1, the IR photon pulse energy directly absorbed in the Co/Pd layer is estimated to be lower than 0.06 μJ. This corresponds to a fluence value within the focus area of 0.06 mJ cm^−2^ (see Methods). Clearly, this amount of energy is more than one order of magnitude too small to trigger any noticeable demagnetization^16^. Within the 40 nm thick Al layer, on the other hand, 10.4 mJ are absorbed.

To analyse the data in more detail, we have renormalized the demagnetization curves to exhibit the same degree of demagnetization. The resulting curves are plotted in Fig. 3(c). Comparing the time traces of the uncapped Co/Pd film with each other (red symbols) one observes for all three the very same initial rapid magnetization decrease. This observation implies that the temporal evolution of this initial demagnetization phase is independent of the specific IR pump fluence. On the other hand, once the magnetization minimum has been reached at about 0.5 ps, the curves exhibit distinctly different dynamics: The partial recovery of the magnetization taking place during this time range is increasingly slowed down with increasing IR pump fluence as it has been observed previously, e.g., in ref. 14,17.

We remark that the fluence independence of the initial demagnetization rate appeared already in our previous demagnetization study, which exploited at a high harmonic generation source the magnetic contrast of the Co M_2,3_ resonance as scattering contrast^14^. Since these M-edges of the transition metals are energetically low-lying (60 eV for Co), it could be argued that the strong, non-resonant contribution of valence electrons might affect these measurements. The replication of these observations obtained here at the Co L_3_ resonance therefore erases any such doubts. We also point out that the absence of any significant variation in the onset of the demagnetization dynamics demonstrates that any timing drift, which may have occurred during this experiment, is correctly accounted for by the optical IR – X-ray pulse cross-correlator.

The renormalized evolution of the capped Co/Pd film is represented by the dark blue circles in Fig. 3(c). The clear “horizontal” separation between the traces of the two films reveals unquestionably the presence of a significant delay between the onset of the respective demagnetization dynamics. As discussed below, we estimate this delay to be about 270 fs. In addition, the comparison reveals a clear difference in the demagnetization rate, which is about a factor of two slower in case of the capped Co/Pd film. These differences show up even more clearly by shifting the curve of the capped film by −380 fs (light blue squares in Fig. 3(c)) so that the minima of the curves for capped and uncapped (weakest pump) films overlap. Interestingly, one notes that the dynamics of the partial recovery of the magnetization of the capped film superposes with the one of the weakly pumped uncapped film (6 μJ). This similarity indicates that about 400 fs after excitation of the magnetization dynamics, the nature of the excitation process itself does not influence the dynamics anymore.

### Data modeling

To characterize the demagnetization rate quantitatively, we have employed the three temperature model to obtain an analytical expression for fitting of the magnetization’s temporal evolution^2^,^16^:


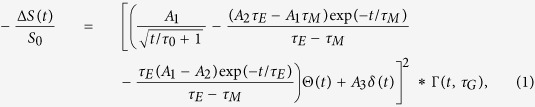


where 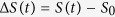 is the variation in scattering intensity, *S*_0_ is the scattering intensity of the unpumped system, *τ*_*M*_ is the demagnetization time, *τ*_*E*_ the time constant characterizing the partial magnetization recovery, and Θ(*t*) the step function. The experimental time resolution enters as Γ(*t*), which we represent as a Gaussian function with a full width at half maximum (FWHM) of *τ*_*G*_. This *τ*_*G*_ is the convolution of the pump and probe pulse width with the uncertainty of the optical IR – X-ray cross-correlator measurement. We remark that prior to the experiment, coarse pump-probe time overlap was determined with a coaxial antenna and this time reference was unchanged during the experiment. The timing of the individual pump-probe events of each time trace was after the experiment corrected with the simultaneously recorded data of the optical IR – X-ray pulse cross-correlator. Finally, to simplify data presentation, we shifted the time axis t_*exp*_ of all curves by the same amount such that *t* = 0 corresponds to pump-probe time overlap of the trace recorded for the uncapped Co/Pd film with low fluence pumping (t_0,*u*_). The time variable in eqn. 1 thus corresponds to 
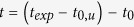
 with *t*_0_ a free fit parameter characterizing the onset of the demagnetization dynamics of the actual data trace. Definition of all other parameters can be found in the literature^2^.

In line with the visual similarity of the initial part of the three demagnetization curves of the uncapped Co/Pd film in Fig. 3(b), the fits of these curves yield within its accuracy identical values for onset (*t*_0_ = 0, see above), demagnetization time (*τ*_*M*_ = 160 fs) and *τ*_*G*_ = 150 fs. We thus fitted these three sets of data with the same time constants. The results are listed in Table 2.

When analyzing the temporal evolution of the capped Co/Pd film, one finds that the demagnetization dynamics can be well-reproduced by the same fit model. In particular this implies that the initial dynamics are correctly reproduced by a single exponential decay function. One notes, however, that this agreement can be reached in two ways: by either increasing the characteristic time scale of the demagnetization process (*τ*_*M*_) or – and – the experimental time resolution (*τ*_*G*_). We can actually understand this inter-dependence within the model put forward by Eschenlohr and co-workers^8^: the strong absorption of IR light by Al leads to the excitation of very hot valence electrons within the top 5–10 nm of the Al cap layer. These thermalize by electron-electron (and also electron-phonon) scattering thus giving rise to a very large number of hot electrons. Interchange between this hot-electron distribution with the colder one of the underlying Co/Pd multilayer acts as trigger for the observed ultrafast demagnetization process as discussed in more detail below.

The conversion of the incident IR laser pulse into a cascade of hot electrons can thus be seen as a temporal stretching of the excitation pulse. Within our fit model this leads to a higher value for the parameter *τ*_*G*_, which combines pump and probe pulse duration with their relative arrival time jitter. It is however not possible to distinguish whether this increase in pump pulse duration is the unique source of the observed slowing down of the demagnetization rate or whether an increase in the time constant of the demagnetization process itself contributes, too. The reason for this is that the only other time scale of relevance here, namely the one of the partial magnetization recovery, is too long to be significantly influenced by a slightly longer pump pulse as attested by the overlap of the uncapped and weakly pumped capped film’s dynamic after the minimum magnetization has been reached (Fig. 3(c)).

In the following, we fix the demagnetization time fit parameter to the value obtained for the uncapped film (*τ*_*M*_ = 160 fs), which is motivated by the absence of any IR fluence dependence of the initial magnetization dynamics of the uncapped film. The obtained fit, shown as solid blue line in Fig. 3(a,b), reproduces the data with excellent precision. For the delay of the onset of the magnetization dynamics it yields a value of *t*_0_ = 270 fs and a time constant *τ*_*E*_ = 1.01 ps for the dynamics of the partial magnetization recovery. As expected from the visual agreement with the uncapped film in case of the lowest pump, this latter value is close to the value of the uncapped film. Finally, *τ*_*G*_ is found to be 430 fs. From this we can derive for the excitation pulse width a value of about 400 fs making the reasonable assumption that neither the X-ray pulse length nor the arrival time correction have changed with respect to the experiment on the capped film realized just minutes before. The main parameters obtained from fitting the experimental data to equation 1 are summarized in Table 2.

## Discussion

The observation of the ultrafast demagnetization dynamics in case of the capped Co/Pd film, for which the IR pump is completely absorbed in the capping layer, demonstrates without doubt that ultrafast demagnetization does not require direct interaction between the electromagnetic pump pulse and the magnetic film. This result therefore resolves the controversy between Eschenlohr *et al.*^8^ and Khorsand *et al.*^9^. Moreover, our observations are in qualitative agreement with the model proposed by Eschenlohr and co-workers^8^, which attributes the photonless excitation of the demagnetization process to the hot electrons generated in the metallic capping layer by absorption of the IR pump pulse. Indeed, the delay between the demagnetization of the capped Co/Pd film compared to the one of the uncapped sample can be seen as the traveling time of the hot electrons through the Al cap layer. And the slowing down of the observed demagnetization dynamics can be understood as a broadening of the excitation pulse due to the stochastic electron-electron scattering cascade. It has to be noted that heat diffusion at the speed of sound (of the order of 5000 ms^−1^ in Al) is at least one order of magnitude too slow to explain our observations.

A detailed calculation of these processes is beyond the scope of this article. We note, however, that the observed delay of the onset of the demagnetization process (*t*_0_ = 270 fs) and the reduced rate of the demagnetization process are qualitatively compatible with the timescales found in previous calculations^3^,^8^. We further remark that in comparison to the values calculated by Eschenlohr *et al.* for their Au (30 nm)/Ni (15 nm)/Al (buffer) trilayer (t_0_ = 70 ± 40 fs and *t*_*M*_ = 400 ± 160 fs)^8^, our larger delay value is in line with our 10 nm thicker cap layer as well as the shorter hot-electron lifetime in our Al layer with respect to the Au layer^18^.

The demagnetization process itself is described within the model of Eschenlohr and co-workers^8^ as consequence of superdiffusive exchange between the initially cold, spin polarized electrons of the ferromagnetic layer and the hot, spin-neutral electrons of the cap layer. Due to the different mobility of spin majority and minority electrons within the ferromagnetic layer, a net transport of magnetic moment out of the ferromagnetic layer occurs into the neighboring non-magnetic, metallic cap and buffer layer. We note that our observations are in agreement with this prediction, however, we do not consider them as providing strong experimental evidence for this model. To obtain such evidence, or even proof, the amount of spin polarization injected from the ferromagnetic layer into the neighboring, non-magnetic layers should be characterized experimentally. Then, a comparison between the amount of lost and gained spin polarization could quantitatively determine the relevance/contribution of superdiffusive spin transport to the overall demagnetization dynamics.

In conclusion, we have demonstrated that ultrafast demagnetization does not require direct excitation of the ferromagnetic material by a photon pulse. In order to prove that affirmation we have measured the magnetization dynamics of a magnetic film capped by a thick Al layer. Contrary to pioneering previous work^8^ the optical properties of this capping layer have been thoroughly characterized and found to strongly absorb the IR pump pulse. The remaining photon pulse reaching the underlying magnetic film is three orders of magnitude too weak to trigger the demagnetization observed. Comparison between the dynamics of this capped film with the ones of an uncapped reference film showed that the onset of the demagnetization dynamics is delayed and the initial loss in magnetization is slowed down. These observations are compatible with a demagnetization process triggered by a flux of hot valence band electrons from the Al cap layer to the underlying magnetic film. The mechanism by which these hot electrons trigger the demagnetization remains to be elucidated.

## Methods

The samples have been grown in an argon atmosphere of 1 × 10^−3^ mbar. The base pressure of the instrument has been 1 × 10^−8^ mbar. The multilayers samples have been deposited on a 2 nm thick Pd seed layer. The deposition rate used were respectively 0.03 nm s^−1^, 0.05 nm s^−1^ and 0.12 nm s^−1^ for Al, Co and Pd. Taking into account the measured transmission of the single Al layer and the theoretical transmission values of the Al/Si_3_N_4_ and Si_3_N_4_/air interfaces (0.24 and 0.89) the absortion in the 40 nm Al layer is estimated to be 
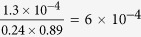
 (note that multiple reflections and interference effects have been neglected within this approximation).

The 800 nm pump pulses triggering the magnetization dynamics were delivered by the femtosecond IR laser of the SXR end station^10^,^19^. To uniformly excite the magnetic film on the 50 μm square membranes, a focusing lens (*f* = 75 cm) was positioned just in front of the beamline’s standard in-coupling mirror, which yields close to collinear beam propagation. The spatial beam profile and position of the IR pump beam was monitored during the experiment using an out-of-vacuum replica of the IR focus. Since the IR pulse length in the SXR hutch is about 50 fs, we chose a comparable X-ray probe pulse length. Both pulse durations are thus below the temporal resolution of the X-ray arrival time jitter correction, which sets the overall time resolution of the experiment to about 130 ± 20 fs^20^.

The bending of the Kirkpatrick-Baez mirrors was set to obtain an X-ray spot size of about 50 μm in diameter comparable to the size of the membranes 50 × 50 μm^2^. The incident X-ray photon flux density (*F*_*X*_) was limited to a value at least 20 times lower than the modification treshold of the magnetic domains structure^21^, i.e. *F*_*X*_ < 2 mJ cm^−2^. One can calculate that of these about 3% are absorbed within the Al cap layer, 50% in the Co/Pd multilayer, 2% in the Pd buffer and 4% in the Si_3_N_4_ membrane. The dynamics is thus safely dominated by the IR excitation.

A particularity of this experiment has been the use of the pnCCD camera^11^ to record single X-ray pulse scattering patterns at the full 120 Hz repetition rate of LCLS. The detector’s central opening adds the possibility to accurately determine the transmitted X-ray intensity with a photodiode and thus to normalize the recorded scattering pattern to the intensity of the photon pulse incident on the spatially constrained Si_3_N_4_ membrane. Within minutes tens of thousands of correctly intensity normalized scattering pattern can thus be recorded. This high data rate can be exploited in an IR pump - X-ray scattering probe experiment by continuously varying the pump-probe delay during data accumulation. We employed for this a random walk scheme centered around pump-probe time overlap and extending towards the desired delay range with decreasing sampling rate. Adding as further ingredient an accurate measure of the individual X-ray arrival time jitter using the optical cross-correlator installed permanently at SXR (timing tool)^19^,^20^, the recorded data are then grouped in time bins matching the desired time resolution of the respective temporal region. The efficiency of this experimental approach is demonstrated by the excellent signal-to-noise ratio of the delay scans shown in Fig. 3(a), which have been recorded within a few minutes each.

## Additional Information

**How to cite this article**: Vodungbo, B. *et al.* Indirect excitation of ultrafast demagnetization. *Sci. Rep.* **6**, 18970; doi: 10.1038/srep18970 (2016).

## Figures and Tables

**Table 1:** Reflectivity, transmission and absorption of the uncapped Co/Pd film (3 nm Al), capped Co/Pd film (40 nm Al), and the 40 nm Al cap itself for 800 nm IR light.

Sample	Reflectivity	Total transmission	Total absorption	Al absorption
3 nm Al capped Co/Pd	0.69	0.01	0.30	0.13
40 nm Al capped Co/Pd	0.84	<10^−6^	0.16	0.16
40 nm Al	0.84	1.3 × 10^−4^	0.16	0.16

Absorption values are derived from the measured reflectivity and transmission data. The sensitivity of the measurement has been 10^−6^ while the relative accuracy is better than 1%. Note that about 1 nm of the Al films is very rapidly oxidized, while further oxidation is slow^23^ and can thus be neglected in view of the short time periods the films have been exposed to air. From the data measured for the 40 nm Al film we derive an IR absorption length of about 5.5 nm, which is in good agreement with the literature.

**Table 2:** Values of the main fit parameters for the uncapped and capped samples.

Sample	*τ*_*M*_ (fs)	*τ*_*E*_ (ps)	*t*_0_ (fs)	*τ*_*G*_ (fs)
Uncapped	160	1.07	0	150
Capped	160	1.01	270	430
