# Corrigendum: Indirect excitation of ultrafast demagnetization

**DOI:** 10.1038/srep21727

**Published:** 2016-03-04

**Authors:** Boris Vodungbo, Bharati Tudu, Jonathan Perron, Renaud Delaunay, Leonard Müller, Magnus H. Berntsen, Gerhard Grübel, Grégory Malinowski, Christian Weier, Julien Gautier, Guillaume Lambert, Philippe Zeitoun, Christian Gutt, Emmanuelle Jal, Alexander H. Reid, Patrick W. Granitzka, Nicolas Jaouen, Georgi L. Dakovski, Stefan Moeller, Michael P. Minitti, Ankush Mitra, Sebastian Carron, Bastian Pfau, Clemens von Korff Schmising, Michael Schneider, Stefan Eisebitt, Jan Lüning

Scientific Reports 6: Article number: 18970; 10.1038/srep18970published online: 01062016; updated: 03042016

The original version of this Article contained a typographical error in the spelling of the author Bharati Tudu, which was incorrectly given as Bahrati Tudu. This has now been corrected in the PDF and HTML versions of the Article.

